# Association of Medicaid Unwinding With Hospital Financial Outcomes Among General Acute Care and Critical Access Hospitals

**DOI:** 10.1111/1475-6773.70182

**Published:** 2026-11-09

**Authors:** Nima Khodakarami, Joel E. Segel

**Affiliations:** ^1^ Department of Health Policy and Administration Penn State University Monaca Pennsylvania USA; ^2^ Center for Applied Studies in Health Economics, Department of Public Health Sciences Penn State College of Medicine Hershey Pennsylvania USA; ^3^ Southwest Rural Health Research Center Texas A&M School of Public Health College Station Texas USA; ^4^ Department of Health Policy and Administration Penn State University University Park Pennsylvania USA; ^5^ Penn State Cancer Institute Hershey Pennsylvania USA; ^6^ Department of Public Health Sciences Penn State College of Medicine Hershey Pennsylvania USA

**Keywords:** critical access hospitals, general acute care hospitals, health insurance, hospital finance, Medicaid unwinding, non‐profit hospitals, uncompensated care

## Abstract

**Objective:**

To examine the association of Medicaid unwinding with hospital financial outcomes (uncompensated care costs and burden, Medicaid revenue, net financial gains, Medicaid operating margin, and total operating margin) among US general acute care (GAC) and critical access (CAH) hospitals.

**Study Setting and Design:**

We used descriptive trend analysis and adjusted hospital‐level fixed‐effects panel regressions to estimate changes in outcomes.

**Data Sources and Analytic Sample:**

National Academy for State Health Policy's (NASHP) Hospital Cost Tool, 2021–2024.

**Principal Findings:**

Uncompensated care costs increased among both GAC ($1.1 M; CI = 0.3–1.9) and CAH ($0.2 M; CI = 0.1–0.3) hospitals and coincided with increases in Medicaid revenue within both GAC and CAH (GAC = $9.1 M; CI = 6.0–12.2 and CAH = $0.8 M; CI = 0.4–1.3) in 2024, however net financial gains (i.e., Medicaid operating profit minus uncompensated care costs) increased only within GAC ($4.9 M; CI = 2.6–7.1). Increases in uncompensated care costs were concentrated among non‐profit hospitals in both GAC and CAH (GAC = $1.7 M; CI = 0.9–2.5 and CAH = $0.2 M; CI = 0.2–0.4). Uncompensated care burden did not increase significantly, while Medicaid revenue, Medicaid operating margins, and total operating margins improved in both hospital types in 2024.

**Conclusions:**

While hospitals experienced increases in their uncompensated care costs following Medicaid unwinding, these increases coincided with rising Medicaid revenue.

## Keypoints

- What is known on this topic?
  ○ During the unwinding of Medicaid continuous enrollment, more than 25 million beneficiaries are being disenrolled nationwide, with wide variation across states.
  ○ Medicaid disenrollment during unwinding was associated with significant disruptions in access to care, with more than half of disenrolled individuals reporting skipped or delayed health care services.
  ○ Following Medicaid unwinding, hospitals began experiencing shifts in payer mix, with early data showing increases in their uninsured population and their median rate of uncompensated care.
- What this study found?
  ○ During Medicaid unwinding, hospital uncompensated care costs and Medicaid revenue increased, with significant increases occurring in fiscal year 2024. Changes in net financial gains were positive for GAC hospitals.
  ○ Among GAC, increases in uncompensated care costs were most pronounced within non‐profit, medium‐sized, and urban hospitals, and those in Medicaid expansion, in the Northeast, and Midwest.
  ○ Among CAH, uncompensated care costs increase broadly distributed in non‐profit, Medicaid expansion and non‐expansion, Midwest and rural hospitals.
  ○ Despite rising uncompensated care costs, the uncompensated care burden did not increase significantly, while Medicaid and total operating margins improved across most GAC and CAH subgroups.

## 1 Introduction

With the introduction of the Families First Coronavirus Response Act in response to the COVID‐19 public health emergency (PHE) in early 2020, states were barred from terminating Medicaid beneficiaries' coverage regardless of fluctuations in their income [1]. After the end of the PHE on March 31, 2023, Medicaid continuous coverage requirements terminated and states were authorized to resume Medicaid eligibility redeterminations. Over an 18‐month period, states reassessed enrollees' eligibility and disenrolled those who no longer qualified for Medicaid coverage. During this “unwinding” process, more than 27 million Medicaid enrollees lost their coverage [2, 3].

Multiple studies documented the results of unwinding, showing evidence of increases in uninsured people following the end of the PHE [4, 5, 6]. These coverage losses varied substantially across states and patient demographics [7, 8], and led to disruptions in access to care [9, 10, 11, 12]. While prior studies also documented shifts in hospitals' payer mix [13, 14], the financial consequences of unwinding to hospitals remain unclear.

Hospital financial outcomes were anticipated to shift during the Medicaid unwinding period through two primary mechanisms. First, Medicaid revenue and associated Medicaid operating margins could decline during the unwinding period as a result of increased rates of individual losing Medicaid coverage [15]. However, the net impact on hospitals' total operating margins will depend on whether patients remain largely uninsured or are able to transition to other forms of insurance (i.e., private insurance in most cases) with higher payment rates [16]. Second, coverage losses during this period could lead to increased uncompensated care costs for uninsured patients [17, 18, 19]. Hospitals are generally unable to collect full payment from uninsured patients, but may fully or partially write‐off these costs through charity care policies [16]. A report from Pennsylvania found that uncompensated care for non‐general acute care hospitals increased by 15.0%, or $2.2 million during the unwinding year of 2023 compared to 2022 [19]. Hence, uncompensated care can negatively affect operating margins from hospitals providing care without corresponding revenue [20, 21, 22].

No prior national study has examined hospital financial outcomes during the Medicaid unwinding period. We examined whether unwinding was associated with changes in hospital finances. This study is important for several reasons. First, uncompensated care cost affects both the generosity of hospitals' existing charity care programs and access to health care [23]. Second, the resulting financial strain may impact the broader community by forcing hospitals to reduce staffing or eliminate essential clinical services [15, 24]. Third, with projected Medicaid cuts [25], it is important to understand the burden of unpaid care on hospitals' financial stability, specifically among rural and safety‐net hospitals, although the unwinding may impact hospital finances differently than proposed broader cuts [26, 27, 28, 29]. This study provides new national evidence that Medicaid unwinding was associated with increases in uncompensated care cost and Medicaid revenue, but no significant change in the burden of uncompensated care, while Medicaid operating profit margins improved.

## 2 Study Data and Methods

We obtained hospital data for 2021 to 2024 from the National Academy for State Health Policy's (NASHP) Hospital Cost Tool (HCT) dashboard [30], which compiles annual financial reports from facilities certified by the Centers for Medicare & Medicaid Services (CMS). These reports are also publicly available through the Healthcare Cost Report Information System (HCRIS). The HCT data have been widely used in analyses of hospital finances [31, 32].

We utilized data from 4633 hospitals nationwide, including 3242 short term general acute care (GAC) and 1391 critical access hospitals (CAH), excluding hospitals in US territories. To obtain a balanced sample of hospitals, we performed several exclusions, which are exhibited in Appendix Figure 1. Our final sample included 3447 hospitals (69% GAC and 31% CAH). Distribution of hospital characteristics are presented in Appendix Table 1. Excluded hospitals were disproportionately for‐profit (37.4%) and small size hospitals with < 100 beds (27.7%).

From HCT data, we used data on hospital's bed size (< 100, 100–299, and ≥ 300 beds), ownership (for‐profit, governmental, nonprofit), and location. We classified hospitals' rurality based on Rural–Urban Commuting Area (RUCA) codes, specifying RUCA 4–10 as rural, and RUCA 1–3 as urban [33]. We further classified hospitals into Medicaid expansion and non‐expansion status based on their state's status as of 2022 [34]. We then categorized hospitals locations as one of four US Census regions. We also used American Community Survey 5‐year estimates to collect county‐level data including patient demographics and economic characteristics for years 2021–2024 [35].

### 2.1 Dependent Variables

We examined six outcomes. The two primary outcomes of interest during the unwinding were *uncompensated care cost*, defined as the sum of charity care cost and uninsured and bad debt cost [24, 36]; and *Medicaid revenue*. We also created a variable of *net financial gains*, as a difference between hospital Medicaid operating profit and uncompensated care costs, to examine hospital financial performance more broadly. The other three outcomes were: (1) *uncompensated care burden*, which provides a sense of the overall burden of uncompensated care that is comparable across hospitals of different sizes and measured as uncompensated care cost divided by the hospital's operating cost [24, 36, 37]; (2) *Medicaid operating profit margin*, measured as Medicaid operating profit divided by Medicaid net patient revenue; and (3) the *hospital's operating profit margin*, reflecting earnings on hospital patient services, excluding non‐patient related income and costs. This is a closely watched marker of financial health across hospitals [38], and is often applied in analyses of hospital finances [22, 39, 40, 41].

### 2.2 Statistical Analysis

First, we provided descriptive trends for primary outcomes from 2021 to 2024 in Figure 1. Second, we estimated adjusted, linear fixed‐effects panel regressions controlling for time‐varying county‐level covariates and clustered standard errors at the state level. The model also includes hospital and year fixed effects. We used 2022 as the pre‐unwinding reference year and estimated changes in outcomes during unwinding in 2023 and 2024. Because mean outcomes across GAC and CAH are significantly different (Appendix Table 2) and given that hospitals' performance may be associated with safety net criteria [22], we performed separate analyses for GAC and CAH. Statistical significance was reported at *p* < 0.05.

**FIGURE 1:**
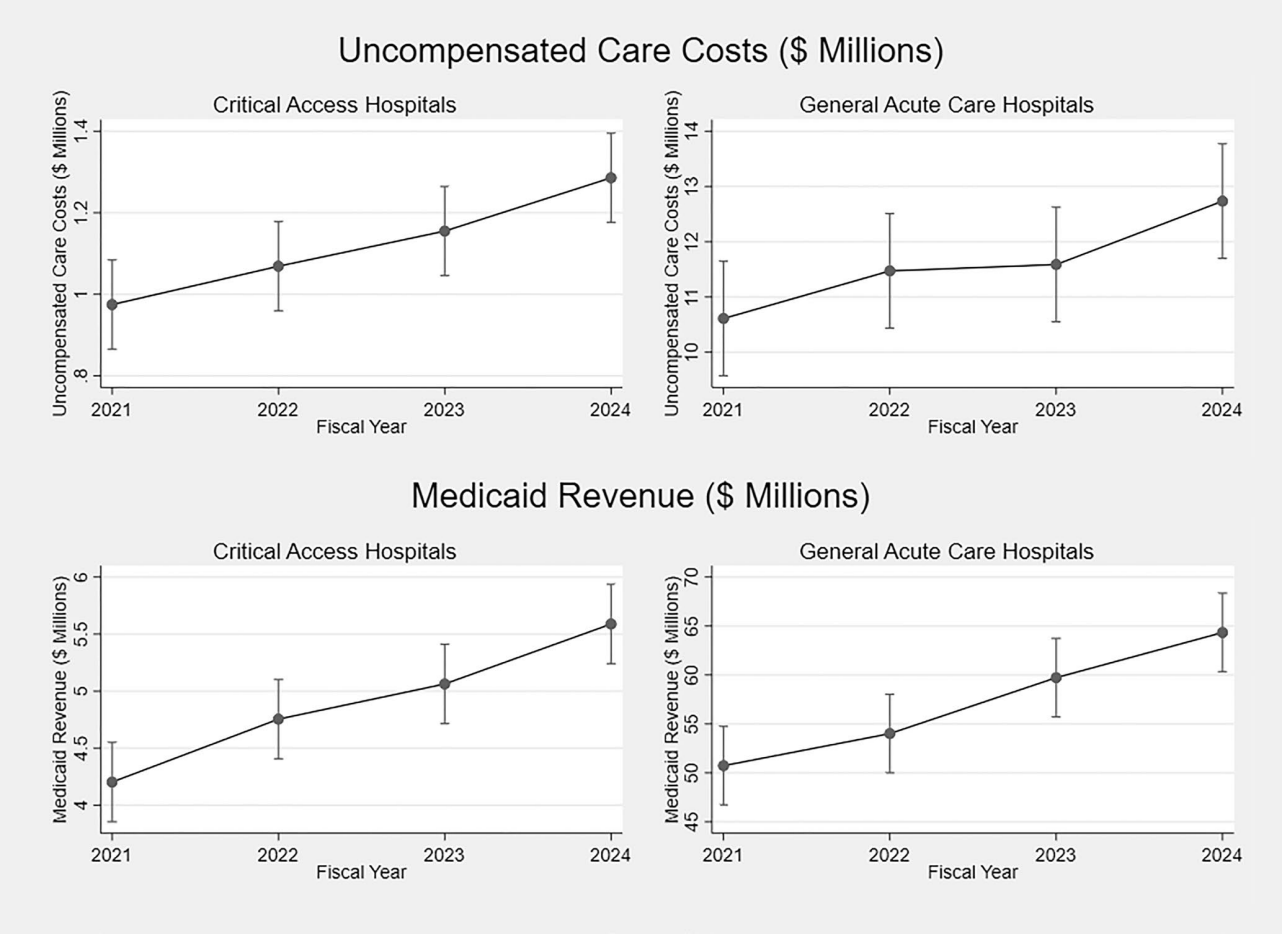
Trends of financial outcomes among general acute care and critical access hospitals between fiscal years 2021–2024. *Source:* Authors' analysis of 2021–2024 National Academy for State Health Policy data. The sample includes 2372 general acute care hospitals and 1075 critical access hospitals nationwide. *Notes:* “Uncompensated care cost” is estimated by aggregating charity care and uninsured and bad debt. “Medicaid Revenue” presents net patient revenue received from the Medicaid programs.

### 2.3 Sensitivity Analyses

We performed several sensitivity analyses. First, we estimated unadjusted regression models to test for potential covariate sensitivity. Second, we conducted subsample analyses by hospital characteristics and their geography to examine heterogeneity in outcomes [22, 42, 43, 44]. Third, we included all hospitals, incorporating the exclusion sample as were described in the Appendix Table 1, in our analysis to address potential sampling bias. Fourth, we estimated log‐transformed models of our primary outcomes to account for skewed Medicaid revenue or uncompensated care costs. Finally, to account for staggered state‐level unwinding start dates and varying state disenrollment aggressiveness, as well as facility‐level dose–response, we estimated an exposure‐intensity model. For each facility‐year observation, we constructed a continuous exposure index combining three elements: (1) the number of months within the hospital's fiscal year exposed to Medicaid unwinding [45], (2) the state‐level Medicaid disenrollment rate from Kaiser Family Foundation (KFF) tracking data [46], and (3) the hospital's pre‐unwinding baseline Medicaid payer mix.

## 3 Study Results

Figure 1 displays mean uncompensated care costs and Medicaid revenue among GAC and CAH hospitals over the study period. For GAC, Medicaid revenue increased from 2021 to 2022 but accelerated significantly post‐2022. For CAH, Medicaid revenue increased steadily from 2021 through 2024. Uncompensated care costs exhibited a continuous upward trend from 2021 to 2024 for both hospital types. These observations are corroborated by regression results in Table 1, which summarizes changes hospital financial outcomes in years 2023 and 2024 relative to the pre‐unwinding year (2022). The adjusted model shows that among GAC, uncompensated care costs increased by nearly $1.2 million (*p* < 0.01) in 2024. Medicaid revenue increased by nearly $5.4 million (*p* < 0.001) and $9.1 million (*p* < 0.001) in 2023 and 2024 respectively. Net financial gains improved by $2.1 million in 2023 (*p* < 0.01) and $4.9 million in 2024 (*p* < 0.001). Despite these changes, the burden of uncompensated care remained unchanged with GAC hospitals. Nevertheless, both Medicaid operating margin (4.5 pp in 2023 and 14.9 pp in 2024; *p* < 0.001) and total operating margin (0.92 pp in 2023 and 3.0 pp %in 2024; *p* < 0.01) increased notably during unwinding as compared to 2022.

**TABLE 1:** Adjusted changes in hospital financial outcomes during the Medicaid unwinding period (2023–2024) relative to 2022.

	Uncompensated care cost ($M)	Medicaid revenue ($M)	Net financial gains ($M)	Uncompens‐ated care burden (pp)	Medicaid operating margin (pp)	Total operating margin (pp)
General acute care hospitals (*N* = 9488)						
Year2023	0.05 (0.23)	5.39*** (1.02)	2.14** (0.67)	−0.19* (0.07)	4.47** (1.47)	0.92** (0.28)
Year2024	1.15** (0.41)	9.11*** (1.53)	4.86*** (1.12)	−0.05 (0.13)	14.92*** (2.10)	3.05*** (0.45)
Critical access hospitals (*N* = 4300)						
Year2023	0.09 (0.05)	0.31*** (0.09)	−0.04 (0.24)	−0.03 (0.24)	−0.21 (1.69)	0.24 (0.56)
Year2024	0.22*** (0.04)	0.84** (0.23)	0.34 (0.21)	0.34 (0.21)	9.85** (3.09)	2.04* (0.86)

*Note:* “Uncompensated care cost” is estimated by aggregating charity care and uninsured and bad debt. “Medicaid Revenue” presents net patient revenue received from the Medicaid programs. “Net financial gains” presents a difference between hospital Medicaid operating profit and uncompensated care costs. “Uncompensated care burden” measured as uncompensated care cost divided by hospital's operating cost. “Medicaid operating margin” indicates Medicaid net patient revenue less Medicaid patient hospital operating costs as a percentage of Medicaid net patient revenue. “Total operating margin” presents the results of net patient revenue minus hospital operating costs divided by net patient revenue, excluding non‐patient related income and costs. N represents hospital‐year observations. The regression‐adjusted model includes year and hospital fixed effects and controls for county‐level covariates. Robust standard errors in parentheses are clustered at the state level.

Abbreviations: $M, millions of dollars; pp, percentage points.

* *p* < 0.05.

** *p* < 0.01.

*** *p* < 0.001.

*Source:* Authors' analysis of 2021–2024 National Academy for State Health Policy data.

Among CAH, uncompensated care costs increased by $0.2 million (*p* < 0.001). Medicaid revenue also increased during the unwinding period, showing $0.3 million increases in 2023 (*p* < 0.001) and $0.8 million in 2024 (*p* < 0.01). However, hospitals' net financial gains and uncompensated care burden didn't change among CAH. Both Medicaid operating margin and total operating margin also experienced significant increases in year 2024, with respectively 9.8 pp (*p* < 0.01) from −33.9% and 2.0 pp (*p* < 0.05) from 3.2 in year 2022.

Appendix Tables 3 and 4 present changes in outcomes across GAC and CAH subgroups. With respect to our primary outcomes, among GAC hospitals, the largest increases in uncompensated care costs were primarily concentrated in 2024 and within the following types of hospitals: non‐profit ($1.69 million, *p* < 0.001), mid‐sized ($1.22 million, *p* < 0.001), urban ($1.59 million, *p* < 0.01), in Medicaid expansion states ($1.59 million, *p* < 0.01), in the Northeast region ($2.92 million, *p* < 0.01), and the Midwest region ($1.45 million, *p* < 0.01). While Medicaid revenue grew significantly across all subgroups in both 2023 and 2024, the largest increases were observed in governmental hospitals ($17.3 million, *p* < 0.001) and large facilities with more than 300 beds ($27.2 million, *p* < 0.001). Among CAH hospitals, the largest increases in uncompensated care costs were concentrated in 2024 and within the following types of hospitals: non‐profit ($0.29 million, *p* < 0.001), rural ($0.21 million, *p* < 0.001), in Medicaid expansion states ($0.23 million, *p* < 0.001), in the Midwest region ($0.25 million, *p* < 0.001), and in the South region ($0.17 million, *p* < 0.05). While Medicaid revenue grew significantly across most subgroups in both 2023 and 2024, the largest statistically significant increases were observed in hospitals in Medicaid expansion states ($1.01 million, *p* < 0.01), governmental hospitals ($0.96 million, *p* < 0.01), and facilities in the West ($1.07 million, *p* < 0.05).

Appendix Tables 5–7 present sensitivity analyses estimates. The direction and magnitude of these estimates corroborated our primary findings, supporting the robustness of the findings across alternative model specifications. Results from the interacted exposure model in Appendix Table 8 revealed only Medicaid revenue increases among GAC in 2024, with no statistically significant changes among CAH in either 2023 or 2024.

## 4 Discussion

This study provides the first national evidence on changes in hospital financial outcomes during the Medicaid unwinding period beginning in 2023. Our findings show a nuanced picture. During unwinding, uncompensated care costs increased among both GAC and CAH; however, these increases were not followed by a proportional rise in uncompensated care burden or a decline in operating margins. Instead, Medicaid revenue and operating margins improved. However, when we accounted for hospital unwinding exposure and state disenrollment pacing, just Medicaid revenue among GAC in 2024 increased significantly.

While the observed increases in uncompensated care costs are consistent with early state‐level reports [19], the improvement in Medicaid revenue may have arisen for several potential reasons. First, the compositional shift in patient mix and per‐capita service intensity rather than total Medicaid enrollment may have contributed to observed changes in Medicaid revenue, namely lower‐cost enrollees, particularly children and healthier adults were disproportionately disenrolled, while sicker, higher‐utilization patients remained on Medicaid (perhaps because they had greater incentive to ensure re‐enrollment) [47]. Alternatively, this rise might just be a return to normal care levels, while care avoidance driven by perceived uninsurance could have suppressed utilization prior to unwinding [4]. This anticipation may also mean the impacts of winding might be quite different from the potential impact of broader Medicaid cuts. Additionally, higher provider payment rates and expanded use of state‐directed payments through managed care organizations may have increased spending per enrollee [47, 48]. Other factors associated with increases in Medicaid revenue may include changes in payment policies or increasing costs of care [49, 50].

The subgroup analyses reveal important heterogeneity that the aggregate findings may obscure. Increases in uncompensated care costs were concentrated among nonprofit hospitals in both GAC and CAH, consistent with their historically greater provision of charity care relative to for‐profit hospitals [51]. Uncompensated care cost increases were also observed within Medicaid expansion states for both hospital types, which was expected given that expansion states accounted for a disproportionate share of Medicaid enrollment and therefore faced greater exposure to eligibility redeterminations during unwinding [46, 52], where approximately 30% of beneficiaries lost coverage, accounting for over 21 million or nearly 78% of total disenrollment [3, 53]. Among GAC, the largest increases in uncompensated care costs were observed among mid‐sized hospitals with 100–299 beds. Among CAH, however, increases were concentrated among rural, and both expansion and non‐expansion states. Rural CAH already operate at negative total operating margins and rising uncompensated care costs compound existing financial pressures. If uncompensated care costs continue to rise, particularly through projected Medicaid cuts [54], rural CAH may be forced to reduce services, cut staffing, or close entirely, with severe consequences for the communities they serve [15]. This concern is amplified by evidence that rural hospitals in non‐expansion states are already less profitable than their expansion‐state counterparts [42], with rising demand for uncompensated care [54].

The widespread improvement in operating margins observed across most hospital subgroups, particularly among GAC, coincided with general price increases [55]. In the absence of sustained price increases, hospitals would likely need to reduce marginal costs associated with safety‐net services [22] or improve operational efficiency to maintain profitability [40]. Hospitals serving large numbers of underinsured or uninsured patients would be most exposed to such reversals and might require additional funding to offset rising uncompensated care costs. Without such support, hospitals with declining profit margins face an elevated risk of closure, merger, acquisition, or conversion to CAH status [38].

These findings offer important policy implications. First, policymakers should monitor uncompensated care trends among nonprofit, rural, and other hospitals that experienced significant rises in uncompensated care costs during the unwinding period. Second, the heterogeneity in CAH outcomes across regions and expansion status suggests that federal and state policymakers should ensure adequate safety‐net protections for the most vulnerable facilities.

This study had several limitations. First, HCRIS data is subject to well‐documented shortcomings, including reporting errors, which may have introduced measurement error [36, 37]. Second, we did not account for changes in state‐level policies, such as variation in uncompensated care payment generosity, which may have influenced outcomes. Third, the consequences of unwinding in each state might be partially offset by other factors, such as the replacement of lost Medicaid revenue with revenues from newly enrolled Medicaid patients, that were not captured in our analysis. Lastly, while our findings document overall financial trends during the unwinding period, causality remains unknown.

While this study documented the early changes in hospital financial outcomes during the Medicaid unwinding, future research should examine these effects over longer time horizons to better assess the implications of sustained coverage losses for hospital financial stability. Additional work is needed to evaluate how state‐level heterogeneity in unwinding implementation contributed to variation in uncompensated care increases, particularly among CAH. Further patient‐ and hospital‐level analyses would improve understanding of how care utilization patterns and hospital finances evolve as Medicaid coverage continues to change.

## Funding

The authors have nothing to report.

## Conflicts of Interest

The authors declare no conflicts of interest.

## Supporting information


**Appendix Table 1.** Descriptive characteristics of hospitals by exclusion status, 2021–2024.
**Appendix Table 2.** Descriptive statistics characteristics of hospitals in year 2022 before Medicaid unwinding.
**Appendix Table 3.** Adjusted changes in hospital financial outcomes during the Medicaid unwinding period (2023–2024) relative to 2022 among *general acute care hospitals*.
**Appendix Table 4.** Adjusted changes in hospital financial outcomes during the Medicaid unwinding period (2023–2024) relative to 2022 among *critical access hospitals*.
**Appendix Table 5.** Unadjusted changes in hospital financial outcomes during the Medicaid unwinding period (2023–2024) relative to 2022.
**Appendix Table 6.** Adjusted changes in financial outcomes among full sample of hospitals during the Medicaid unwinding period (2023–2024) relative to 2022.
**Appendix Table 7.** Log‐transformed estimates of changes in financial outcomes among hospitals during the Medicaid unwinding period (2023–2024) relative to 2022.
**Appendix Table 8.** Adjusted changes in hospital financial outcomes during the Medicaid unwinding period (2023–2024) relative to 2022 by Medicaid unwinding exposure intensity.
**Appendix Figure 1.** Sample selection and exclusion diagram.
**Appendix Figure 2.** Trend of financial outcomes among *general acute care hospitals* between years 2021–2024.
**Appendix Figure 3.** Trend of financial outcomes among *critical access hospitals* between years 2021–2024.
**Appendix Figure 4.** Estimated changes in financial outcomes relative to 2022 among *general acute care hospitals*.
**Appendix Figure 5.** Estimated changes in financial outcomes relative to 2022 among *critical access hospitals*.

## Data Availability

The data that support the findings of this study are available in National Academy for State Health Policy. Hospital Cost Tool at https://tool.nashp.org/. These data were derived from the following resources available in the public domain: National Academy for State Health Policy. Hospital Cost Tool, https://tool.nashp.org/.
