# Supplementary material for: Association of Medicaid Unwinding With Hospital Financial Outcomes Among General Acute Care and Critical Access Hospitals
Source: Health Serv Res. 2026 Nov 9;61(6):e70182. doi: 10.1111/1475-6773.70182 (PMC13747709; doi:10.1111/1475-6773.70182)
Supplement: Supplementary file 1 [file HESR-61-e70182-s001.docx]

Appendix

**Table 1:** Descriptive characteristics of hospitals by exclusion status, 2021–2024

|  | **All Hospitals** | | | **General Acute Care Hospitals** | | | **Critical Access Hospitals** | | |
| --- | --- | --- | --- | --- | --- | --- | --- | --- | --- |
|  | Total | Exclude | Include | Total | Exclude | Include | Total | Exclude | Include |
| **Total** | 4,633 | 1,186 | 3,447 | 3,242 | 870 | 2,372 | 1,391 | 316 | 1,075 |
| **Ownership Type** (%) | | | | | | | | | |
| For-Profit | 816  (17.6%) | 305  (25.7%) | 511  (14.8%) | 757  (23.3%) | 280  (32.2%) | 477  (20.1%) | 59  (4.2%) | 25 | 34 |
| Governmental | 1,036 (22.4%) | 288  (24.3%) | 748  (21.7%) | 486  (15.0%) | 159  (18.3%) | 327  (13.8%) | 550  (39.5%) | 129 | 421 |
| Non-Profit | 2,781 (60.0%) | 593  (50.0%) | 2,188  (63.5%) | 1,999  (61.7%) | 431  (49.5%) | 1,568  (66.1%) | 782  (56.2%) | 162 | 620 |
| **Bed Size** (%) | | | | | | | | | |
| <100 | 2,580  (55.7%) | 714  (60.2%) | 1,866  (54.1%) | 1,221  (37.7%) | 430  (49.4%) | 791  (33.3%) | 1,391  (100%) | 316  (100%) | 1,075  (100%) |
| >100-299 | 1,262  (27.2%) | 236  (19.9%) | 1,026  (29.8%) | 1,262  (38.9%) | 236  (27.1%) | 1,026  (43.3%) | 0 | 0 | 0 |
| ≥ 300 | 663  (14.3%) | 108  (9.1%) | 555  (16.1%) | 663  (20.5%) | 108  (12.4%) | 555  (23.4%) | 0 | 0 | 0 |
| **Rurality** (%) | | | | | | | | | |
| Urban | 2,393  (51.7%) | 595  (50.2%) | 1,798  (52.2%) | 2,282  (70.4%) | 570  (65.5%) | 1,712  (72.2%) | 111  (8.0%) | 25  (7.9%) | 86  (8.0%) |
| Rural | 2,240  (48.3%) | 591  (49.8%) | 1,649  (47.8%) | 960  (29.6%) | 300  (34.5%) | 660  (27.8%) | 1,280  (92.0%) | 291  (92.1%) | 989  (92.0%) |
| **Medicaid Expansion** (%) | | | | | | | | | |
| Expansion | 3,139  (66.8%) | 774  (65.3%) | 2,365  (68.6%) | 2,160  (66.6%) | 564  (64.8%) | 1,596  (67.3%) | 979  (70.4%) | 210  (66.5%) | 769  (71.5%) |
| Non-Expansion | 1,494  (32.2%) | 412  (34.7%) | 1,082  (31.4%) | 1,082  (33.4%) | 306  (35.2%) | 776  (32.7%) | 412  (29.6%) | 106  (33.5%) | 306  (28.5%) |
| **Census Regions** (%) | | | | | | | | | |
| Northeast | 560  (12.1%) | 118  (9.9%) | 442  (12.8%) | 482  (14.9%) | 106  (12.2%) | 376  (15.9%) | 78  (5.6%) | 12  (3.8%) | 66  (6.1%) |
| Midwest | 1,385  (29.9%) | 286  (24.1%) | 1,099  (31.9%) | 747  (23.0%) | 166  (19.1%) | 581  (24.5%) | 638  (45.9%) | 120  (38.0%) | 518  (48.2%) |
| South | 1,749  (37.8%) | 524  (44.2%) | 1,225  (35.5%) | 1,379  (42.5%) | 415  (47.7%) | 964  (40.6%) | 370  (26.6%) | 109  (34.5%) | 261  (24.3%) |
| West | 939  (20.3%) | 258  (21.8%) | 681  (19.8%) | 634  (19.6%) | 183  (21.0%) | 451  (19.0%) | 305  (21.9%) | 75  (23.7%) | 230  (21.4%) |

*Source:* Authors analysis of 2021-2024 National Academy for State Health Policy data. Details of hospital exclusions/inclusion are exhibited in the Appendix Figure 1. Percentages are calculated within each of the nine columns, using that column's total number of hospitals as the denominator.

**Table 2:** Descriptive statistics characteristics of hospitals in year 2022 before Medicaid unwinding

|  | General Acute Care Hospitals  (*N=*2,372) | Critical Access Hospitals  (*N=*1,075) | *p-*value |
| --- | --- | --- | --- |
| Uncompensated care cost ($ million) | 11.47 | 1.07 | *p*<0.001 |
| Medicaid revenue ($ million) | 54.01 | 4.75 | *p*<0.001 |
| Net financial gains ($ million) | -18.86 | -1.73 | *p*<0.001 |
| Uncompensated care burden (%) | 4.20 | 3.58 | *p*<0.001 |
| Medicaid operating margin (%) | -31.54 | -33.97 | *P*= 0.307 |
| Total operating margin (%) | 12.38 | 3.19 | *p*<0.001 |

*Source:* Authors analysis of 2021-2024 National Academy for State Health Policy data. *Notes:* “Uncompensated care cost” is estimated by aggregating charity care and uninsured and bad debt. “Medicaid Revenue” presents net patient revenue received from the Medicaid programs. “Net financial gains” presents a difference between hospital Medicaid operating profit and uncompensated care costs. “Uncompensated care burden” measured as uncompensated care cost divided by hospital’s operating cost. “Medicaid operating margin” indicates Medicaid net patient revenue less Medicaid patient hospital operating costs as percentage of Medicaid net patient revenue. “Total operating margin” presents the results of net patient revenue minus hospital operating costs divided by net patient revenue, excluding non-patient related income and costs. All statistics in the table are the mean for a hospital year pre-Medicaid unwinding.

**Table 3:** Adjusted changes in hospital financial outcomes during the Medicaid unwinding period (2023-24) relative to 2022 among *general acute care hospitals*

|  | Uncompe-nsated care cost ($M) | Medicaid revenue ($M) | Net financial gains ($M) | Uncompens-ated care burden (pp) | Medicaid operating margin (pp) | Total operating margin (pp) |
| --- | --- | --- | --- | --- | --- | --- |
| **Ownership Type** | | | | | | |
| For-Profit (*N=*1,908) | | | | | | |
| $Year 2023$ | 0.20  (0.13) | 2.34***  (0.62) | 2.10**  (0.71) | -0.24**  (0.07) | 6.32  (3.29) | 0.03  (0.75) |
| $Year 2024$ | 0.38  (0.26) | 5.27**  (1.60) | 5.05***  (1.19) | -0.23*  (0.11) | 20.29***  (4.49) | 1.46  (1.24) |
| Governmental (*N=*1,308) | | | | | | |
| $Year 2023$ | -0.48  (0.48) | 7.93***  (2.02) | 3.79  (2.12) | -0.51*  (0.23) | 4.11  (4.53) | 0.49  (1.08) |
| $Year 2024$ | -0.14  (1.17) | 17.32***  (3.24) | 11.40***  (2.58) | -0.56*  (0.21) | 14.47***  (3.63) | 1.48  (1.24) |
| Non-Profit (*N=*6,272) | | | | | | |
| $Year 2023$ | 0.26  (0.26) | 5.78***  (1.21) | 1.81*  (0.76) | -0.09  (0.06) | 3.98**  (1.28) | 1.29***  (0.24) |
| $Year 2024$ | 1.69***  (0.41) | 8.63***  (1.71) | 3.47*  (1.5) | 0.13  (0.13) | 13.36***  (1.78) | 3.89***  (0.42) |
| **Bed Size** | | | | | | |
| <100 beds (*N=3,164)* | | | | | | |
| $Year 2023$ | 0.005  (0.06) | 0.89***  (0.20) | 0.31  (0.22) | -0.27*  (0.14) | 1.51  (2.18) | 0.112  (0.42) |
| $Year 2024$ | 0.20*  (0.09) | 1.76***  (0.36) | 1.44***  (0.32) | -0.17  (0.17) | 14.41***  (2.08) | 2.41***  (0.68) |
| 100-299 beds (*N=4,104)* | | | | | | |
| $Year 2023$ | 0.34*  (0.14) | 3.05***  (0.77) | 1.62***  (0.46) | -0.06  (0.07) | 6.36***  (1.78) | 1.15**  (0.39) |
| $Year 2024$ | 1.22***  (0.27) | 5.80***  (0.98) | 4.05***  (0.85) | 0.09  (0.12) | 15.55***  (2.49) | 3.23***  (0.58) |
| ≥ 300 beds (*N=2,220)* | | | | | | |
| $Year 2023$ | -0.28  (0.91) | 16.58***  (3.09) | 5.90*  (2.51) | -0.25*  (0.09) | 4.84*  (2.03) | 1.63**  (0.46) |
| $Year 2024$ | 2.66  (1.39) | 27.25***  (4.60) | 12.08**  (4.49) | -0.06  (0.14) | 14.17***  (3.34) | 3.67***  (0.55) |
| **Rurality** | | | | | | |
| Urban (*N=6,848)* | | | | | | |
| $Year 2023$ | 0.12  (0.31) | 7.12***  (1.33) | 2.77**  (0.89) | -0.13*  (0.06) | 6.17***  (1.64) | 1.40***  (3.36) |
| $Year 2024$ | 1.59**  (0.54) | 11.69***  (2.01) | 5.72***  (1.49) | 0.06  (0.11) | 16.75***  (2.54) | 3.35***  (0.53) |
| Rural (*N=2,640)* | | | | | | |
| $Year 2023$ | -0.09  (0.09) | 1.06**  (0.32) | 0.63*  (0.31) | -0.30*  (0.15) | 0.32  (2.05) | -0.19  (0.52) |
| $Year 2024$ | 0.05  (0.17) | 2.68***  (0.74) | 2.75***  (0.67) | -0.28  (0.21) | 10.48***  (2.54) | 2.42**  (0.85) |
| **Medicaid Expansion** | | | | | | |
| Expansion (*N=6,384)* | | | | | | |
| $Year 2023$ | 0.29  (0.29) | 6.39***  (1.32) | 1.82*  (0.79) | -0.09  (0.09) | 4.61**  (1.46) | 0.62*  (0.31) |
| $Year 2024$ | 1.59**  (0.51) | 11.14***  (1.68) | 3.89**  (1.36) | 0.28*  (0.14) | 12.12***  (2.01) | 2.56***  (0.53) |
| Non-Expansion (*N=3,104)* | | | | | | |
| $Year 2023$ | -0.45  (0.32) | 3.59**  (1.12) | 2.83*  (1.24) | -0.38**  (0.10) | 4.07  (3.29) | 1.43*  (0.55) |
| $Year 2024$ | 0.18  (0.78) | 5.21*  (2.21) | 6.67**  (1.99) | -0.71*  (0.23) | 20.31***  (4.52) | 3.89***  (0.82) |
| **Census Regions** | | | | | | |
| Northeast (*N=1,504)* | | | | | | |
| $Year 2023$ | 1.05*  (0.38) | 8.85  (3.90) | 0.43  (1.39) | -0.03  (0.06) | 2.88  (4.04) | 0.94*  (0.33) |
| $Year 2024$ | 2.92**  (0.76) | 12.70*  (4.59) | -2.37  (1.76) | 0.34*  (0.10) | 10.95*  (4.77) | 3.82**  (0.81) |
| Midwest (*N=2,324)* | | | | | | |
| $Year 2023$ | 0.08  (0.36) | 3.24  (1.66) | 2.04  (1.59) | -0.04  (0.13) | 6.84*  (2.52) | 0.99**  (0.26) |
| $Year 2024$ | 1.45*  (0.51) | 6.81*  (2.44) | 5.17*  (2.04) | 0.17  (0.21) | 15.25**  (1.36) | 2.29*  (0.85) |
| South (*N=3,856)* | | | | | | |
| $Year 2023$ | -0.43  (0.32) | 5.34**  (1.40) | 3.35*  (1.24) | -0.44**  (0.12) | 3.66  (2.65) | 1.58**  (0.53) |
| $Year 2024$ | 0.54  (0.68) | 7.38**  (2.29) | 7.02**  (1.86) | -0.43  (0.24) | 16.92***  (3.98) | 3.87***  (0.70) |
| West (*N=1,804)* | | | | | | |
| $Year 2023$ | 0.40  (0.43) | 5.14*  (2.14) | 0.66  (0.52) | 0.09  (0.10) | 3.32  (2.16) | -0.74  (0.39) |
| $Year 2024$ | 0.88  (0.66) | 12.80***  (2.03) | 5.02*  (2.12) | 0.22  (0.18) | 11.81*  (4.74) | 1.49  (0.80) |

*Source:* Authors analysis of 2021-2024 National Academy for State Health Policy data. *Notes:* “Uncompensated care cost” is estimated by aggregating charity care and uninsured and bad debt. “Medicaid Revenue” presents net patient revenue received from the Medicaid programs. “Net financial gains” presents a difference between hospital Medicaid operating profit and uncompensated care costs. “Uncompensated care burden” measured as uncompensated care cost divided by hospital’s operating cost. “Medicaid operating margin” indicates Medicaid net patient revenue less Medicaid patient hospital operating costs as percentage of Medicaid net patient revenue. “Total operating margin” presents the results of net patient revenue minus hospital operating costs divided by net patient revenue, excluding non-patient related income and costs. The regression-adjusted model includes year and hospital fixed effects and controls for county-level covariates. N represents hospital-year observations. Robust standard errors in parentheses are clustered at the state level. $M = millions of dollars. pp = percentage points. *P<0.05 **P<0.01 ***P<0.001.

**Table 4:** Adjusted changes in hospital financial outcomes during the Medicaid unwinding period (2023-24) relative to 2022 among *critical access hospitals*

|  | Uncompe-nsated care cost ($M) | Medicaid revenue ($M) | Net financial gains ($M) | Uncompens-ated care burden (pp) | Medicaid operating margin (pp) | Total operating margin (pp) |
| --- | --- | --- | --- | --- | --- | --- |
| **Ownership Type** | | | | | | |
| For-Profit (*N=136*) | | | | | | |
| $Year 2023$ | 0.21  (0.18) | -0.12  (0.27) | -0.82*  (0.34) | -0.10  (0.72) | -5.16  (10.37) | -9.00*  (3.74) |
| $Year 2024$ | 0.21  (0.15) | 1.36  (0.85) | 0.78  (0.82) | -0.86  (0.58) | 21.43  (20.87) | -3.99  (4.17) |
| Governmental (*N=1,684*) | | | | | | |
| $Year 2023$ | -0.03  (0.08) | 0.35***  (0.09) | 0.05  (0.08) | -0.47  (0.49) | -0.63  (2.74) | -0.11  (0.58) |
| $Year 2024$ | 0.12  (0.07) | 0.96**  (0.28) | 0.64*  (0.28) | 0.26  (0.40) | 12.99**  (3.64) | 1.30  (1.08) |
| Non-Profit (*N=2,480*) | | | | | | |
| $Year 2023$ | 0.17**  (0.5) | 0.31*  (0.12) | -0.16  (0.17) | 0.27  (0.16) | 0.16  (2.00) | 1.00  (0.79) |
| $Year 2024$ | 0.29***  (0.04) | 0.76**  (0.26) | 0.29  (0.26) | 0.43*  (0.17) | 7.44*  (3.39) | 2.88**  (0.98) |
| **Bed Size** | | | | | | |
| <100 beds (*N=4,300*) | | | | | | |
| $Year 2023$ | 0.09  (0.05) | 0.31***  (0.09) | -0.09  (0.10) | -0.03  (0.24) | -0.21  (1.69) | 0.24  (0.56) |
| $Year 2024$ | 0.22***  (0.04) | 0.84**  (0.23) | 0.44  (0.24) | 0.34  (0.21) | 9.85**  (3.09) | 2.04*  (0.86) |
| **Rurality** | | | | | | |
| Urban (*N=344*) | | | | | | |
| $Year 2023$ | 0.16  (0.13) | 0.61  (0.31) | 0.15  (0.37) | -0.34  (0.31) | 2.74  (4.23) | 1.92  (3.61) |
| $Year 2024$ | 0.37  (0.14) | 0.91*  (0.37) | 0.50  (0.42) | 0.16  (0.27) | 12.02  (5.98) | 3.89  (3.87) |
| Rural (*N=3,956*) | | | | | | |
| $Year 2023$ | 0.08  (0.05) | 0.29**  (0.09) | -0.11  (0.11) | -0.01  (0.25) | -0.35  (1.66) | 0.09  (0.45) |
| $Year 2024$ | 0.21***  (0.04) | 0.84**  (0.24) | 0.44  (0.25) | 0.35  (0.23) | 9.75**  (3.24) | 1.89*  (0.75) |
| **Medicaid Expansion** | | | | | | |
| Expansion (*N=3,076*) | | | | | | |
| $Year 2023$ | 0.05  (0.05) | 0.39***  (0.11) | -0.01  (0.13) | -0.12  (0.19) | -1.42  (2.07) | 0.27  (0.72) |
| $Year 2024$ | 0.23***  (0.05) | 1.01**  (0.31) | 0.56  (0.32) | 0.35  (0.19) | 9.95*  (3.83) | 2.63*  (1.07) |
| Non-Expansion (*N=1,224*) | | | | | | |
| $Year 2023$ | 0.19  (0.15) | 0.10  (0.09) | -0.31  (0.14) | 0.22  (0.74) | 2.29  (3.06) | 0.10  (0.84) |
| $Year 2024$ | 0.19*  (0.07) | 0.40*  (0.14) | 0.14  (0.15) | 0.29  (0.59) | 11.91*  (4.58) | 0.45  (1.16) |
| **Census Regions** | | | | | | |
| Northeast (*N=264*) | | | | | | |
| $Year 2023$ | 0.23  (0.12) | 0.14  (0.34) | -0.73  (0.51) | 0.19  (0.26) | -1.89  (11.46) | -1.15  (1.03) |
| $Year 2024$ | 0.25  (0.13) | 0.55  (0.51) | -0.07  (0.58) | -0.08  (0.14) | 2.66  (14.09) | 0.47  (2.22) |
| Midwest (*N=2,072*) | | | | | | |
| $Year 2023$ | 0.16*  (0.05) | 0.22*  (0.07) | -0.22  (0.10) | 0.44  (0.21) | -1.57  (1.99) | -0.36  (0.53) |
| $Year 2024$ | 0.25***  (0.05) | 0.72  (0.39) | 0.30  (0.40) | 0.78  (0.31) | 9.67  (5.31) | 2.37*  (1.08) |
| South (*N=1,044*) | | | | | | |
| $Year 2023$ | -0.08  (0.13) | 0.35  (0.24) | 0.24  (0.25) | -1.01  (0.69) | 2.25  (3.16) | 1.69  (1.97) |
| $Year 2024$ | 0.17*  (0.08) | 0.93  (0.52) | 0.67  (0.48) | 0.26  (0.46) | 10.34*  (4.78) | 2.35  (2.68) |
| West (*N=920*) | | | | | | |
| $Year 2023$ | 0.10  (0.09) | 0.49  (0.25) | -0.01  (0.28) | 0.10  (0.28) | -0.07  (4.13) | 0.44  (0.37) |
| $Year 2024$ | 0.21  (0.15) | 1.07*  (0.36) | 0.60  (0.43) | 0.32  (0.34) | 11.22  (5.30) | 1.58  (0.97) |

*Source:* Authors analysis of 2021-2024 National Academy for State Health Policy data. *Notes:* “Uncompensated care cost” is estimated by aggregating charity care and uninsured and bad debt. “Medicaid Revenue” presents net patient revenue received from the Medicaid programs. “Net financial gains” presents a difference between hospital Medicaid operating profit and uncompensated care costs. “Uncompensated care burden” measured as uncompensated care cost divided by hospital’s operating cost. “Medicaid operating margin” indicates Medicaid net patient revenue less Medicaid patient hospital operating costs as percentage of Medicaid net patient revenue. “Total operating margin” presents the results of net patient revenue minus hospital operating costs divided by net patient revenue, excluding non-patient related income and costs. The regression-adjusted model includes year and hospital fixed effects and controls for county-level covariates. N represents hospital-year observations. Robust standard errors in parentheses are clustered at the state level. $M = millions of dollars. pp = percentage points. *P<0.05 **P<0.01 ***P<0.001.

**Table 5:** Unadjusted changes in hospital financial outcomes during the Medicaid unwinding period (2023-24) relative to 2022

|  | Uncompens-ated care cost ($M) | Medicaid revenue ($M) | Net financial gains ($M) | Uncompens-ated care burden (pp) | Medicaid operating margin (pp) | Total operating margin (pp) |
| --- | --- | --- | --- | --- | --- | --- |
| General Acute Care Hospitals *(N=*9,488*)* | | | | | | |
| $Year 2023$ | 0.11  (0.24) | 5.71***  (1.01) | 2.11**  (0.68) | -0.19**  (0.07) | 4.79**  (1.60) | 0.98***  (0.29) |
| $Year 2024$ | 1.26**  (0.42) | 10.32***  (1.64) | 5.19***  (1.23) | -0.04  (0.13) | 15.29***  (2.06) | 3.20***  (0.46) |
| Critical Access Hospitals (*N=*4300) | | | | | | |
| $Year 2023$ | 0.09  (0.05) | 0.31***  (0.09) | -0.10  (0.09) | -0.08  (0.23) | -0.41  (1.70) | 0.37  (0.55) |
| $Year 2024$ | 0.22***  (0.04) | 0.83**  (0.24) | 0.45  (0.25) | 0.26  (0.19) | 9.95**  (3.12) | 2.21**  (0.82) |

*Source:* Authors analysis of 2021-2024 National Academy for State Health Policy data. *Notes:* “Uncompensated care cost” is estimated by aggregating charity care and uninsured and bad debt. “Medicaid Revenue” presents net patient revenue received from the Medicaid programs. “Net financial gains” presents a difference between hospital Medicaid operating profit and uncompensated care costs. “Uncompensated care burden” measured as uncompensated care cost divided by hospital’s operating cost. “Medicaid operating margin” indicates Medicaid net patient revenue less Medicaid patient hospital operating costs as percentage of Medicaid net patient revenue. “Total operating margin” presents the results of net patient revenue minus hospital operating costs divided by net patient revenue, excluding non-patient related income and costs. The regression model includes year and hospital fixed effects. N represents hospital-year observations. Robust standard errors in parentheses are clustered at the state level. $M = millions of dollars. pp = percentage points. *P<0.05 **P<0.01 ***P<0.001.

**Table 6:** Adjusted changes in financial outcomes among full sample of hospitals during the Medicaid unwinding period (2023-24) relative to 2022

|  | Uncompens-ated care cost ($M) | Medicaid revenue ($M) | Net financial gains ($M) | Uncompens-ated care burden (pp) | Medicaid operating margin (pp) | Total operating margin (pp) |
| --- | --- | --- | --- | --- | --- | --- |
| General Acute Care Hospitals (*N=*12,164) | | | | | | |
| $Year 2023$ | 0.10  (0.27) | 5.52***  (0.99) | 2.49**  (0.66) | -0.14*  (0.06) | 4.72**  (1.45) | 1.20***  (0.24) |
| $Year 2024$ | 1.17*  (0.46) | 9.42***  (1.56) | 5.45***  (1.12) | -0.06  (0.12) | 14.71***  (1.98) | 3.01***  (0.53) |
| Critical Access Hospitals (*N=*5,382) | | | | | | |
| $Year 2023$ | 0.08  (0.05) | 0.20*  (0.08) | -0.16  (0.09) | 0.02  (0.21) | -1.53  (1.70) | 0.24  (0.54) |
| $Year 2024$ | 0.22***  (0.04) | 0.78***  (0.22) | 0.35  (0.23) | 0.20  (0.18) | 9.87***  (2.69) | 2.10**  (0.77) |

*Source:* Authors analysis of 2021-2024 National Academy for State Health Policy data. *Notes:* “Uncompensated care cost” is estimated by aggregating charity care and uninsured and bad debt. “Medicaid Revenue” presents net patient revenue received from the Medicaid programs. “Net financial gains” presents a difference between hospital Medicaid operating profit and uncompensated care costs. “Uncompensated care burden” measured as uncompensated care cost divided by hospital’s operating cost. “Medicaid operating margin” indicates Medicaid net patient revenue less Medicaid patient hospital operating costs as percentage of Medicaid net patient revenue. “Total operating margin” presents the results of net patient revenue minus hospital operating costs divided by net patient revenue, excluding non-patient related income and costs. The regression-adjusted model includes year and hospital fixed effects and controls for county-level covariates. N represents hospital-year observations. Robust standard errors in parentheses are clustered at the state level. $M = millions of dollars. pp = percentage points. *P<0.05 **P<0.01 ***P<0.001.

**Table 7:** Log-transformed estimates of changes in financial outcomes among hospitals during the Medicaid unwinding period (2023-24) relative to 2022

|  |  | | Uncompensated  care cost | | Medicaid revenue | | Net financial gains | |
| --- | --- | --- | --- | --- | --- | --- | --- | --- |
|  | General Acute Care Hospitals *(N=9,488)* | | | | | | | |
| $Year 2023$ | | 0.03  (0.02) | | 0.07***  (0.01) | | 0.25***  (0.05) | |  |
| $Year 2024$ | | 0.11***  (0.03) | | 0.12***  (0.02) | | 0.55***  (0.11) | |  |
|  | Critical Access Hospitals *(N=4,300)* | | | | | | | |
| $Year 2023$ | | 0.03  (0.02) | | 0.04***  (0.01) | | -0.02  (0.03) | |  |
| $Year 2024$ | | 0.09***  (0.01) | | 0.10**  (0.03) | | 0.16  (0.09) | |  |

*Source:* Authors analysis of 2021-2024 National Academy for State Health Policy data. *Notes:* “Uncompensated care cost” is estimated by aggregating charity care and uninsured and bad debt. “Medicaid Revenue” presents net patient revenue received from the Medicaid programs. “Net financial gains” presents a difference between hospital Medicaid operating profit and uncompensated care costs. The regression model includes year and hospital fixed effects and controls for county-level covariates. N represents hospital-year observations. Robust standard errors in parentheses are clustered at the state level. Outcome variables are log-transformed. Point estimates represent log point changes; multiplying by 100 provides approximate percentage changes. *P<0.05 **P<0.01 ***P<0.001.

**Table 8:** Adjusted changes in hospital financial outcomes during the Medicaid unwinding period (2023-24) relative to 2022 by Medicaid unwinding exposure intensity

|  |  | | Uncompe-nsated care cost ($M) | | Medicaid revenue  ($M) | | Net financial gains  ($M) | | Uncompe-nsated care burden (pp) | | Medicaid operating margin (pp) | | Total operating margin (pp) | |
| --- | --- | --- | --- | --- | --- | --- | --- | --- | --- | --- | --- | --- | --- | --- |
|  | General Acute Care Hospitals (*N=*9,488) | | | | | | | | | | | | | |
| $2023 exposure$ | | 0.01  (0.03) | | -0.05  (0.20) | | 0.10  (0.17) | | 0.001  (0.01) | | 0.52  (0.33) | | 0.09  (0.05) | |  |
| $2024 exposure$ | | -0.12  (0.08) | | 0.97***  (0.15) | | 0.14  (0.27) | | 0.004  (0.03) | | 0.08  (0.38) | | 0.05  (0.08) | |  |
|  | Critical Access Hospitals (*N=*4,300) | | | | | | | | | | | | | |
| $2023 exposure$ | | -0.01  (0.01) | | 0.03  (0.02) | | 0.05  (0.03) | | 0.01  (0.06) | | 0.42  (0.42) | | 0.05  (0.09) | |  |
| $2024 exposure$ | | 0.01  (0.01) | | 0.05  (0.06) | | -0.03  (0.06) | | 0.05  (0.05) | | -0.41  (0.56) | | -0.11  (0.16) | |  |

Source: Authors analysis of 2021-2024 National Academy for State Health Policy data. . The regression model includes year and hospital fixed effects and controls for county-level covariates. N represents hospital-year observations. Robust standard errors in parentheses are clustered at the state level. $M = millions of dollars. pp = percentage points. *P<0.05 **P<0.01 ***P<0.001.

**Figure 1:** Sample selection and exclusion diagram

Source: Authors analysis of 2021-2024 National Academy for State Health Policy data.

**Figure 2:** Trend of financial outcomes among *general acute care hospitals* between years 2021-2024

Source: Authors analysis of 2021-2024 National Academy for State Health Policy data. The sample includes 2,372 general acute care hospitals nationwide. “Net financial gains” presents a difference between hospital Medicaid operating profit and uncompensated care costs. “Uncompensated care burden” measured as uncompensated care cost divided by hospital’s operating cost. “Medicaid operating margin” indicates Medicaid net patient revenue less Medicaid patient hospital operating costs as percentage of Medicaid net patient revenue. “Total operating margin” presents the results of net patient revenue minus hospital operating costs divided by net patient revenue, excluding non-patient related income and costs.

**Figure 3:** Trend of financial outcomes among *critical access hospitals* between years 2021-2024

Source: Authors analysis of 2021-2024 National Academy for State Health Policy data. The sample includes 1,075 critical access hospitals nationwide. “Net financial gains” presents a difference between hospital Medicaid operating profit and uncompensated care costs. “Uncompensated care burden” measured as uncompensated care cost divided by hospital’s operating cost. “Medicaid operating margin” indicates Medicaid net patient revenue less Medicaid patient hospital operating costs as percentage of Medicaid net patient revenue. “Total operating margin” presents the results of net patient revenue minus hospital operating costs divided by net patient revenue, excluding non-patient related income and costs.

**Figure 4:** Estimated changes in financial outcomes relative to 2022 among *general acute care hospitals*

Source: Authors analysis of 2021-2024 National Academy for State Health Policy data. The sample includes 2,372 general acute care hospitals nationwide. Notes: “Uncompensated care cost” is estimated by aggregating charity care and uninsured and bad debt. “Medicaid Revenue” presents net patient revenue received from the Medicaid programs. “Net financial gains” presents a difference between hospital Medicaid operating profit and uncompensated care costs. “Uncompensated care burden” measured as uncompensated care cost divided by hospital’s operating cost. “Medicaid operating margin” indicates Medicaid net patient revenue less Medicaid patient hospital operating costs as percentage of Medicaid net patient revenue. “Total operating margin” presents the results of net patient revenue minus hospital operating costs divided by net patient revenue, excluding non-patient related income and costs.

**Figure 5:** Estimated changes in financial outcomes relative to 2022 among *critical access hospitals*

Source: Authors analysis of 2021-2024 National Academy for State Health Policy data. The sample includes 1,075 critical access hospitals nationwide. Notes: “Uncompensated care cost” is estimated by aggregating charity care and uninsured and bad debt. “Medicaid Revenue” presents net patient revenue received from the Medicaid programs. “Net financial gains” presents a difference between hospital Medicaid operating profit and uncompensated care costs. “Uncompensated care burden” measured as uncompensated care cost divided by hospital’s operating cost. “Medicaid operating margin” indicates Medicaid net patient revenue less Medicaid patient hospital operating costs as percentage of Medicaid net patient revenue. “Total operating margin” presents the results of net patient revenue minus hospital operating costs divided by net patient revenue, excluding non-patient related income and costs.
